# Community based participatory research approaches to combat oral health inequities among American Indian and Alaska Native populations

**DOI:** 10.1111/jphd.12525

**Published:** 2022-06-21

**Authors:** Carolyn Camplain, Christine Kirby, Steven D. Barger, Heather Thomas, Marissa Tutt, Kristan Elwell, Sara Young, Gerlinda Morrison, Stephanie Hyeoma, Julie A. Baldwin

**Affiliations:** ^1^ Center for Health Equity Research Northern Arizona University Flagstaff Arizona USA; ^2^ Department of Psychological Sciences Northern Arizona University Flagstaff Arizona USA; ^3^ Department of Dental Hygiene Northern Arizona University Flagstaff Arizona USA; ^4^ Department of Health Sciences Northern Arizona University Flagstaff Arizona USA; ^5^ Little Big Horn College Crow Agency Montana USA; ^6^ Hopi Department of Health and Human Services Kykotsmovi Montana USA

**Keywords:** American Indian/Alaska Native, community based participatory research, early childhood caries, indigenous health, oral health

## Abstract

American Indian and Alaska Native (AI/AN) communities have experienced a history of systemic racism and still face significant oral health disparities. These disparities extend to the youngest community members in the form of early childhood caries (ECC). Although behavior and biology contribute to ECC, the conditions where people live, grow, and work, and the systems and political and economic forces that shape individual health outcomes, are thought to greatly impact ECC among AI/AN populations. To address ECC in AI/AN communities, we used a community based participatory approach that incorporated social determinants of health. We found that implementing culturally‐tailored, culturally‐centered, and AI/AN‐created materials for ECC interventions is viewed favorably by community members and tribal leaders. Because of the complexity of ECC in AI/AN communities we adopted a bundled approach of best practices to reduce ECC including: (1) incorporating locally, contextually, and culturally relevant strategies to present recommended ECC prevention approaches; (2) employing AI/AN community members as educators; (3) utilizing motivational interviewing with expectant mothers; and (4) providing fluoride varnish. Our work underscores the importance of developing trusting partnerships with each other and with our communities, drawing upon the insights of community advisory board members, and eliciting formative assessment data from tribal members to gain a more holistic understanding of our participants' lived experience to design relevant intervention materials. Incorporating local knowledge and situating Western oral health prevention approaches within culturally aligned frameworks can enhance partnerships and create sustainable materials for community work.

American Indian and Alaska Native (AI/AN) communities have experienced a history of systemic racism and in this context experience significant oral health disparities. These disparities extend to the youngest community members in the form of early childhood caries (ECC). ECC is the presence of one or more decayed, missing, or filled primary teeth in children younger than 6 years old [1]. AI/AN children suffer disproportionally from ECC, with 62.3% of 2–5 year‐old AI/AN children experiencing caries compared to 42% of Mexican American, 32% of African American, and 25% of non‐Hispanic white preschoolers [2]. Children with ECC are often not seen for treatment until disease has progressed, presenting with pain, inflammation and/or infection [3]. Treatment delay often results in additional sequelae such as eating and sleeping dysfunction, poorer nutrition, and impaired physical development, which can adversely impact families and caregivers [3].

Although behaviors (oral hygiene and dietary habits) and biology (i.e., cariogenic bacteria and genetic factors) may contribute to ECC [4], the conditions where people live, grow, and work, and the systems and political and economic forces that shape individual health outcomes [5, 6], are thought to greatly impact ECC among AI/AN populations. These social determinants of oral health include low oral health literacy and less access to oral health care, as well as less access to other health resources such as clean water and healthful foods [5, 6]. Contributors to oral health disparities among AI/AN communities include distance to health care providers, lack of transportation, shortage of dental providers and clinics, high cost of oral health care, fear of dentists, and other concerns that subordinate attention to oral health care [7]. In addition, oral health care and educational materials are not created by or tailored to AI/AN communities.

To address AI/AN ECC disparities we developed and initiated an ECC prevention program in two AI communities in the western US. This began with a formative assessment to understand the prevention context [7]. This assessment involved interviewing dentists, dental hygienists, public health nurses, mothers/pregnant women and other non‐maternal caregivers in both communities. Interviews assessed resources for and barriers to promoting children's oral health, whether child oral health is a priority for caregivers, and caregiver knowledge about the association between oral health and dietary practices. The formative assessment informed study planning efforts, highlighting specific areas of emphasis for ECC prevention. Community representatives were collaborative partners from the outset. Additionally, community representatives helped liaise with tribal leadership, coordinating input and approval from tribal authorities for potential intervention approaches. This initial scoping laid the groundwork for development of culturally tailored materials.

Prior work demonstrates that culturally‐tailored, culturally‐centered, and traditional Indigenous education approaches are favorably received in AI/AN populations. Culturally‐centered takes culture into account in how health communication is practiced. It attempts to modify the existing health communication practices to suit the characteristics of the culture [8]. It also emphasizes community power to confront social determinants of health [9]. For example, Heaton and colleagues used storytelling to address oral health knowledge in California AI/AN communities. This approach was generally well received, but community members also expressed misgivings regarding their ability to enact the oral health messages because of local barriers [10]. Despite these challenges, this message modality reflects Indigenous models of education and is a mechanism for communicating core values in the community.

Our ECC prevention study used elements of a community based participatory (CBPR) approach that incorporated social determinants of health. Broadly, CBPR links researchers with communities to solve health and social issues through shared problem‐solving and capacity‐building [11]. Principles of CBPR include working within the community to enhance collaboration, building relationships, and expanding efforts over time, allowing for community ownership and sustainability [11]. CBPR honors the contribution of all partners and ensures that collaboration benefits all involved [11]. Our study employed Community Health Representatives (CHRs) to assist with the development of oral health care messages that align with local norms and practices in both communities. They, along with our Community Advisory Board and other community partners, reviewed study materials and provided formal and informal guidance on oral health practices and community health goals [7]. Because CHRs work with the local health infrastructure and share the language, ethnicity, and life experiences of the communities, they maximize the “fit” of prevention materials with the local context [12].

Our study created culturally‐tailored, culturally‐centered, and AI/AN‐created materials for each of our community partner sites. For example, we commissioned original artwork from community‐member artists and solicited photos from community members and CHRs to use for educational and recruitment materials (i.e., study posters). To tailor and focus on our sites, we implemented feedback from our pilot study by removing images of non‐Native individuals and replaced them with images of Native peoples from our communities recommended by the CAB. Feedback from our CAB also allowed us to replace mainstream American food images with locally relevant foods. Further, we followed feedback altering text to more locally relevant goals for mothers (i.e., we changed “taking a class” to “learning to bead.”). We developed oral health education materials that included information about mother's oral health, taking your child to the dentist, and germs causing cavities, with an eye for actions that would be relevant for children up to 3 years of age. We anticipate that these culturally aligned oral health materials will be shared through local social networks, increasing the likelihood of impactful healthcare messaging.

Overlaying this effort were intervention choices driven by prior work in Indigenous communities. The intervention adopted a bundled approach of best practices to reduce ECC. These included: (1) incorporating locally, contextually, and culturally relevant strategies to present recommended ECC prevention approaches; (2) employing AI/AN community members (CHRs) as educators to implement the intervention; (3) utilizing motivational interviewing to educate mothers about ECC during pregnancy and through the child's first 3 years of life; and (4) providing preventive treatment via fluoride varnish application.

While each of these best practices has some evidence of effectiveness, it appears that they need to be combined to prevent ECC in Indigenous communities. For example, applying fluoride varnish was not effective in reducing caries among indigenous children aged 3–5 years [13] and motivational interviewing in the absence of fluoride varnish was not effective despite beginning the intervention earlier, that is, with mother/newborn dyads [14]. However, ECC was reduced in Indigenous children when using motivational interviewing with expectant mothers and when fluoride varnish was applied early, when children were 6–18 months of age [15]. This combination of motivational interviewing, fluoride varnish and treatment initiation in pregnancy resulted in benefits 5 years after the treatment [16]. Paralleling this approach, we integrated western medical approaches (fluoride varnish) with culturally relevant and tailored materials (OH curriculum) and adapted (MI) approaches delivered by community member CHRs. Although fluoride varnish has been shown to be an effective tool to prevent and treat ECC [17, 18], our formative assessment interviews and discussions with our Community Advisory Board and community partners uncovered some hesitation and misinformation regarding fluoride. This included overall fear of fluoride and concern that fluoride varnish was dangerous and fluoridation in the water meant contamination. However, our Community Advisory Boards, community partners, and CHRs were supportive of the use of fluoride varnish and because of its clinical effectiveness, fluoride varnish application was included in both arms of our study. As for MI, it has been culturally tailored [19] and utilized in other trials to combat ECC [14].

When creating interventions for AI/AN communities, materials and practices should be centered on applicability and relevance to participants. Interventions should allow for sustainable practices (practices that last beyond close of study) with meaningful educational materials. Working with both Community Advisory Boards, a pilot of the intervention, and community partners at both sites informed the development of our curriculum materials. Utilizing the tribal members' knowledge of community, culture, and resources was a strength to create relevant education materials that would pair with the MI. Each site was able to develop their own materials related to their goals and practices in daily life.

Our work has taught us the importance of developing trusting partnerships with each other and with our communities, drawing upon the insights of community advisory board members, and eliciting formative assessment data from tribal members to gain a more holistic understanding of our participants' lived experience to design relevant intervention materials. Deferring to local knowledge instead of simply reinforcing Western oral health knowledge can enhance partnerships and create sustainable materials for community work. Though standards of care and best practices for the prevention of ECC are well‐established, there are unique challenges and barriers to achieving optimal oral health among AI/AN communities that must be addressed through using CBPR research approaches and culturally‐centered oral health interventions. These partnerships reflect and honor the communities they serve and in doing so align with culturally appropriate approaches to oral health education and practice that are inclusive of Indigenous knowledge systems.

## CONFLICT OF INTEREST

The authors declare no potential conflict of interest.

## INSTITUTIONAL REVIEW BOARD

The Northern Arizona University Institutional Review Board approved this study on April 11th 2019 (IRB #1396150).
